# Assessing the Influence of Climate Change and Environmental Factors on the Top Tick-Borne Diseases in the United States: A Systematic Review

**DOI:** 10.3390/microorganisms12010050

**Published:** 2023-12-27

**Authors:** Gargi Deshpande, Jessica E. Beetch, John G. Heller, Ozair H. Naqvi, Katrin Gaardbo Kuhn

**Affiliations:** Department of Biostatistics & Epidemiology, Hudson College of Public Health, University of Oklahoma Health Sciences Center, Oklahoma City, OK 73104, USA; gargi-deshpande@ouhsc.edu (G.D.); jessica-beetch@ouhsc.edu (J.E.B.); john-heller@ouhsc.edu (J.G.H.); ozair-naqvi@ouhsc.edu (O.H.N.)

**Keywords:** tick-borne disease, vector-borne disease, United States, Lyme disease, rickettsioses, Rocky Mountain spotted fever, anaplasmosis, climate change, One Health

## Abstract

In the United States (US), tick-borne diseases (TBDs) have more than doubled in the past fifteen years and are a major contributor to the overall burden of vector-borne diseases. The most common TBDs in the US—Lyme disease, rickettsioses (including Rocky Mountain spotted fever), and anaplasmosis—have gradually shifted in recent years, resulting in increased morbidity and mortality. In this systematic review, we examined climate change and other environmental factors that have influenced the epidemiology of these TBDs in the US while highlighting the opportunities for a One Health approach to mitigating their impact. We searched Medline Plus, PUBMED, and Google Scholar for studies focused on these three TBDs in the US from January 2018 to August 2023. Data selection and extraction were completed using Covidence, and the risk of bias was assessed with the ROBINS-I tool. The review included 84 papers covering multiple states across the US. We found that climate, seasonality and temporality, and land use are important environmental factors that impact the epidemiology and patterns of TBDs. The emerging trends, influenced by environmental factors, emphasize the need for region-specific research to aid in the prediction and prevention of TBDs.

## 1. Introduction

Ticks are responsible for transmitting almost 95% of vector-borne diseases (VBDs), such as parasites, bacteria, and viruses, reported in the United States (US) [1]. Out of the nearly 30 different tick-borne diseases (TBDs) identified in the Western Hemisphere, 12 are currently considered existing or emerging threats to human health in the US [2]. Of these, the three most frequently reported are Lyme disease (LD), rickettsioses (including Rocky Mountain spotted fever, or RMSF), and anaplasmosis. In recent years, there has been a gradual shift in the patterns of emerging and re-emerging VBDs, including those transmitted by ticks. This shift can be attributed to several factors that contribute to the increased risk of human exposure to arthropod vectors, resulting in a higher transmission of pathogens to populations that may not have any prior immunity [3,4].

In the United States, all human infections of known TBDs are mandatorily notifiable to local public health authorities, and state-wide distributions and case incidences are collected and reported through the Centers for Disease Control and Prevention (CDC). LD is caused by the bacterium *Borrelia burgdorferi* and is the most commonly reported VBD in the US, with 34,949 confirmed cases in 2019 [5,6]. The bacterium is transmitted to humans through the bite of an infected blacklegged tick, *Ixodes scapularis*. In the past two decades, this species has expanded its geographical distribution within the US, populating new habitats and regions [7]. RMSF is another high-burden TBD caused by *Rickettsia rickettsii* that, if left untreated, can cause widespread vasculitis, resulting in multiple organ failure and death. RMSF transmission is primarily endemic in the Eastern, Central, and Western US to correlate with the distribution of its tick vector, the American dog tick (*Dermacentor variabilis*) [8]. The third most important TBD in the US is anaplasmosis, caused by the bacterium *Anaplasma phagocytophilum*. Cases of *A. phagocytophilum* infections in humans have been recorded in North America, Europe, and Asia, with a more than 16-fold increase in human cases reported in the US since 2000 [9]. All TBDs in the US are zoonotic, which means that in addition to humans, their transmission also relies on other animal hosts, often wild or domesticated mammals. In the case of LD, several wildlife species are important hosts for *I. scapularis* and act as natural reservoirs for *B. burgdorferi,* such as white-tailed deer (*Odocoileus virginianus*), white-footed mice (*Peromyscus leucopus*), and migratory songbirds [10]. Blood meal frequency and reservoir competence vary between these wildlife hosts, and the dispersal of the pathogen through the long-distance movements of these wildlife hosts may play a key role in ongoing range expansions for both the tick and pathogen [10]. A lower efficiency in transmission between white-footed mice and ticks, in addition to a narrower range of vertebrate reservoir hosts, has led to the delayed expansion of *B. microti*, which is also transmitted by *I. scapularis* [11]. Eastern Arizona and northwestern Mexico have experienced an emergence of RMSF vectored by brown dog ticks over the last two decades [12,13]. Parasitism by brown dog ticks and the transmission risk of *R. rickettsii* are associated with an increase in free-roaming dogs and the presence of highly infested dogs in the environment [14,15]. These relationships highlight the significance of adopting a One Health strategy and implementing extended-term epidemiological research to gain a deeper insight into the transmission mechanisms and the interactions among the pathogens, vectors, and hosts [16].

Land use changes are an important factor believed to facilitate the continued spread of TBDs in the US. In the Eastern US, historical land use trends exhibited a decline in agriculture followed by reforestation after the abandonment of cropland, pasture, and cleared lands in the 19th and early 20th centuries [17]. The expansion of forests into previous agricultural areas and, more recently, into urban areas like urban forests or greenspaces is believed by some researchers to be a crucial driver in the emergence of TBDs. They suggest this because *I. scapularis* ticks are closely associated with deciduous forests [18]. Conversely, other researchers have pointed to forest fragmentation and the decline in biodiversity as the primary factors behind the emergence of TBDs [19].

Tick activity is highly dependent on the ambient climate. In the northeastern region of the US, LD is typically transmitted to humans during the late spring and summer [20]. Transmission usually occurs when people come into contact with questing nymph-stage blacklegged ticks while participating in outdoor activities. Various weather conditions, such as temperature [21], precipitation levels [22], and humidity [23], can affect the abundance and behavior of ticks, thereby impacting their development, survival, and activity. These factors also influence human behavior, including the likelihood of engaging in outdoor recreational activities [24]. Notably, the period when nymph-stage ticks are active coincides with a significant period of increased outdoor human activity. It is difficult to determine the exact impact of each factor involved in transmission, but evidence lends to the belief that the combination of small nymph size and their activity aligning with the time when humans spend more time outdoors during late spring and summer contributes to the increased occurrence of TBDs in humans during that period [25].

The direct impact of climate change on TBDs has been demonstrated and projected globally. A recent study on the prevalence of LD in Slovenia, a country that experiences a high burden of LD, showed a strong link between climate changes and spatial expansion in an existing LD focus. The study also showed a 10% increase in the risk of LD infection at the end of the current century when considering five significantly different global climate models [26]. However, LD projections in the US are predicted to increase by 20% in the coming decades, assuming a 2 °C increase in annual average temperatures in line with mid-century (2036–2065) projections from the latest US National Climate Assessment [27].

While it is recognized that environmental factors such as temperature and humidity play a role in affecting the occurrence of LD, their influence on the microbial community within ticks remains largely unexplored [28]. Research has indicated that the endosymbiotic bacteria present in ticks play vital functions, including impacting reproductive fitness [29], providing nutrients [30], and influencing the acquisition, virulence, and transmission of pathogens [31]. A study examined the bacterial microbiome of laboratory-reared *I. scapularis* ticks, which were incubated at various temperatures (4, 20, 30, and 37 °C) under constant humidity conditions, by comparing sequenced bacterial genes with those from untreated baseline controls in a controlled laboratory environment. The investigation revealed alterations in the bacterial microbiome of male *I. scapularis* ticks following static incubation in a controlled laboratory environment at 30 °C for over a week and 37 °C for more than 5 days while maintaining a constant humidity level of over 80%. Furthermore, male ticks subjected to incubation at 30 and 37 °C showed significant variations in bacterial diversity (Shannon index) in comparison to the baseline population, and the changes in diversity were observed to be dependent on the duration of exposure. The findings demonstrated that the bacterial microbiome of *I. scapularis* ticks can be influenced by the environmental temperature in a laboratory environment. Future studies focussing on environmental variables influencing vector microbiome composition and the possible effect of this on the ticks’ ability to carry and transmit pathogens are crucial to understanding the impact of climate change on the risk and spread of tick-borne and other zoonotic diseases [28].

Forecasting the occurrence of LD, anaplasmosis, or RMSF with the aim of informing disease prevention and control strategies is a complex task [32]. The primary challenge associated with forecasting is that the involved tick populations vary from one area to another, across states, and even within a singular county each year. Moreover, factors such as healthcare provider awareness and the methods used for testing and reporting influence the reported cases of TBDs. Regardless of the challenges involved in diagnosis, approximately half a million individuals receive diagnoses and treatment for TBDs annually. Every year presents a significant challenge in managing these diseases [9,32]. 

Reducing human exposure to TBDs is commonly believed to be achievable by controlling the size of tick populations. Researchers exploring various methods have identified chemical and biological substances that prove lethal to ticks, such as synthetic pyrethroids, organophosphates, and entomopathogenic fungi [33]. Field trials consistently demonstrate that the application of acaricides, whether chemical or biological, can effectively reduce tick populations by 50–90% [34,35]. Additionally, the combination of acaricides with other interventions, such as wildlife and landscape management, has been examined. However, the evaluation of integrated approaches to ascertain their effectiveness in reducing human exposure to ticks is hindered by design limitations. These limitations include researchers not being blinded to treatment assignments, the absence of appropriate placebo controls, limited deployment scales, imbalanced designs, and inadequate statistical power. Moreover, studies generally lack data on human health outcomes, particularly the incidence of TBDs [36,37].

Given the current status of the literature on the subject, it is challenging to delineate factors contributing to the spread of TBDs because of the complexities listed, but identifying evidence through research can form a foundation for preventing and predicting TBD incidences. This foundation is warranted given the current burden of disease and the potential for significant increases in such burdens in the future. In this manuscript, we review published papers and papers in pre-print (medRxiv) to collect evidence on influential environmental determinants of TBDs and the effectiveness of preventive strategies in modifying the incidence of TBDs in the US.

## 2. Materials and Methods

The protocol for this systematic review was developed in accordance with the 2020 Preferred Reporting Items for Systematic Reviews and Meta-Analyses (PRISMA) guidelines. The PROSPERO code is 486115.

### 2.1. Eligibility Criteria

Studies were selected according to the following criteria:

1. Geographic location: studies were included if they focused on TBDs within the US, irrespective of the region within the country.
2. Exposures: the included studies had to report on environmental factors that may influence TBDs, including but not limited to climate variables (temperature, precipitation, humidity), agricultural practices, land use changes, landscape ecology, direct human–tick or human–host interactions, and the impact of preventive measures.
3. Outcomes: the primary outcomes of interest were the incidence and prevalence of LD, rickettsioses (including RMSF), and anaplasmosis.
4. Study design: acceptable designs included systematic and/or narrative reviews, retrospective cohort studies, analyses of local or national surveillance data, and modeling studies that addressed the review question.
5. Language: only studies published in English were included due to the language’s predominance in scientific communication in the US.

### 2.2. Exclusion Criteria

Studies were excluded for the following reasons:

1. Outcomes: studies that did not measure the incidence or prevalence of the diseases of interest (LD, rickettsioses, and anaplasmosis) were excluded.
2. Geographic location: studies conducted outside of the US were excluded.
3. Exposures: studies that did not focus on the relevant environmental, agricultural, or land use settings as they pertain to TBDs were excluded.
4. Study design: editorials, commentaries, case reports, case series, and cross-sectional studies were excluded.
5. Language: papers that were not published in English were excluded.

All of our criteria were rigorously applied to ensure that the review focused on pertinent, high-quality studies.

### 2.3. Search Strategy

We searched Medline Plus, PUBMED, and Google Scholar for studies published or submitted for medRxiv pre-print between 1 January 2018 and 28 August 2023. We began the literature search timeline in 2018 because of the significant increase in reported LD cases in the US in 2017, reaching the highest count since 1992 [32]. We used the following search terms: “Tick-borne disease”, “Lyme disease”, “Borrelia burgdorferi”, “Rocky Mountain spotted fever”, “rickettsioses”, “anaplasmosis”, “climate change”, “environmental factors”, “land use”, “One Health”, and “United States”. We used Boolean operators (AND, OR) to combine search terms. The search strategy was peer-reviewed by a reference specialist, and the search was executed on 30 August 2023.

### 2.4. Result Screening and Selection

We collated and uploaded our initially identified records into Covidence, which was utilized to remove duplicates. Two independent reviewers screened the titles and abstracts against the inclusion criteria. Full-text articles were retrieved for further assessment where necessary. Any reviewer discrepancies were resolved through discussion or adjudication by a third reviewer.

### 2.5. Data Extraction and Synthesis

Data were extracted by two independent reviewers using a standardized data collection form that we customized on Covidence. The extracted data included the study title, study aims, study type, start and end dates, and the key findings relevant to the review question. Any discrepancies in data extraction were resolved through discussion or, if necessary, by a third reviewer. We also performed a descriptive synthesis due to the anticipated variability in study designs and outcomes.

### 2.6. Risk of Bias Assessment

Two reviewers independently appraised the methodological quality of the included studies using relevant bias domains and scoring categories from the ROBINS-I tool for non-randomized studies of interventions [38]. We assessed bias across five domains: confounding, selection of participants in the study, measurement of exposures or outcomes, missing data, and selection of studies or reported outcomes. The scoring categories for each domain were low risk, moderate risk, and high risk of bias. Any disagreements were resolved by consensus or by involving a third reviewer.

### 2.7. Data Management

All identified records, screening decisions, and the rationale for exclusion at the full-text stage were recorded in Covidence, a web-based software platform for systematic review and data management [39]. We also used Covidence for data extraction and quality assessments.

## 3. Results

**Figure 1:**
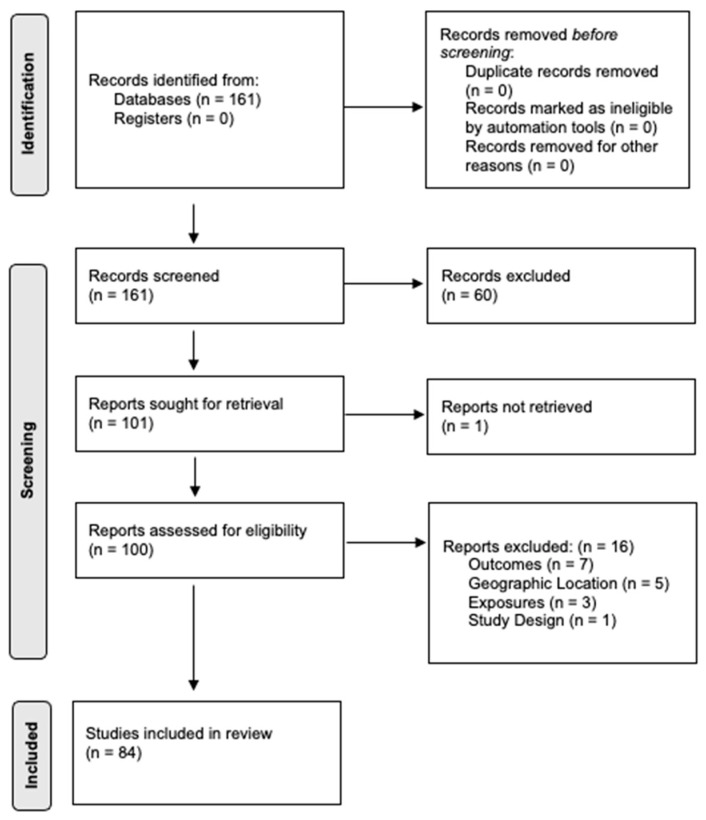
PRISMA 2020 flow diagram of study selection.

**Figure 2:**
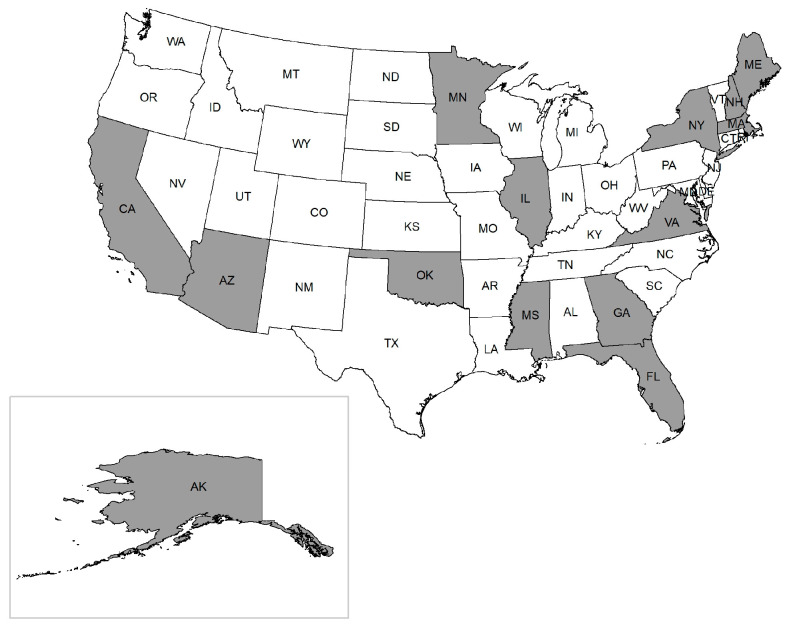
Map of states included in the systematic review, where shaded states represent those included.

We identified 3033 articles published and in pre-print through our preliminary search for LD, rickettsioses, anaplasmosis, and their multiple determinants. Out of the total papers found, we retained 161 for title and abstract screening, given our predefined inclusion and exclusion criteria. Ultimately, 84 papers were included in the final review (Figure 1). The studies were conducted in different regions across the US, with the highest number of studies conducted in New York State (11%). The other states covered in the review include California, Florida, Georgia, Illinois, Maine, Arizona, Massachusetts, Mississippi, Minnesota, New Hampshire, Oklahoma, Virginia, and Alaska (Figure 2). Among the three broad categories of determinants, the papers addressed climate parameters most (32.1%), followed by the impact of land use or landscape factors (23.8%), surveillance studies (16.6%), interventions and prevention strategies (11.9%), environmental factors (10.7%), and tick behavior and tick–host interactions (4.8%). The detailed results of the data extraction are outlined in Supplementary Material, Table S1.

### 3.1. Risk of Bias Assessment

We assessed the methodological quality and risk of bias in the studies using relevant domains from the ROBINS-I tool. Our evaluation encompassed four domains: confounding, the measurement of exposures or outcomes, missing data, and the selection of reported outcomes. Among the 84 studies, the assessment of the risk of bias revealed the following: 90.48% of studies demonstrated a low risk in ‘bias due to confounding’; 88.10% in ‘bias in measurements of exposures or outcomes (information bias)’; 91.67% in ‘bias due to missing data (selection bias)’; and 92.86% in ‘bias in selection of studies or reported outcomes (selection bias)’. We found a moderate bias for 18 studies in the ‘bias in selection of studies or reported outcomes’.

### 3.2. Climate

The complex ecology of VBDs makes it challenging to estimate the impact of climate change and the resulting alteration in the magnitude and geographic distribution of TBDs. A few previous studies have attempted to delineate the true impact of climate change on TBDs, but it remains unclear how these effects translate into TBD incidence and how broadly they apply to a local region [40]. Adverse health impacts from climate change are related to the direct consequences of temperature and precipitation patterns, such as heat stroke and flood injuries, or indirect effects, such as changes in the growing seasons for allergen sources and an expanding vector population [41]. Continued trends for warmer winters and more flooding suggest a much greater risk for the expansion and virulence of a number of VBDs [42]. Additionally, these climate changes can influence the behavior of disease-carrying ticks and their inclination towards choosing a host. In an experiment aiming to measure the impact of temperature on host choice in *Rhipicephalus sanguineus*, which is a potential vector for rickettsioses, the authors demonstrated that as temperatures rise, the likelihood of adult ticks biting humans increases. Among the temperate subspecies of *Rh. sanguineus*, elevated temperatures led to a greater inclination toward selecting humans over dogs. Although a similar number of ticks made a host choice at each temperature, a higher proportion opted for humans in warmer conditions. In contrast, among the tropical subspecies, higher temperatures resulted in an overall increase in the total number of ticks choosing humans [43,44].

The effect of climate is particularly apparent in the case of LD, as it is the most common vector-borne disease in the temperate zone of the US [45]. A study reported the projected changes in LD rates for the time periods of 2040 to 2050 and 2090 to 2100 compared to the previously observed rates from 2010 to 2020. These projections were made under two distinct climate scenarios, one representing the upper range of the literature consensus on emissions and one representing a more moderate emissions mitigation scenario. In the context of the most extreme climate change projection, it is anticipated that the northeast will experience a substantial rise in LD cases, while no regions were projected to experience a significant increase or decrease in the moderate climate change scenario. By the years 2040 to 2050, the number of cases is projected to increase by 23,619 ± 21,607 and 61,776 ± 27,578 by 2090–2100 [40]. One study developed species distribution models using more than 600 geocoded tick records from 51 Californian counties to determine the habitat suitability for western black-legged ticks (*Ixodes pacificus*). The probability of classifying a county as suitable gradually rises as temperatures exceed 0 °C during the coldest quarter of the year. Furthermore, when precipitation falls within the range of 400 mm to 800 mm during the same period, it results in a further increase in suitability. This suggests that areas in California experiencing relatively warm and wet winters are the more appropriate habitats for *I. pacificus* [46]. Another study conducted in California investigated how environmental suitability for tick host-seeking changes seasonally and found that the seasonal suitability for adult *I. pacificus* ticks varied substantially between the different future climate scenarios. Forecasts for the majority of ecoregions in northern California indicate that there is a projected rise in average suitability for host-seeking activities during the winter months for the hotter and drier scenarios modelled. However, this situation is significantly different when considering wetter future scenarios in northern California, where the average suitability during the winter months notably decreases and the duration of seasonal activity may shorten by as much as two months. Overall, these results suggest that in locations where the current conditions are comparatively hot and dry, precipitation or moisture availability may be key limiting factors for seasonal adult *I. pacificus* questing [47].

### 3.3. Seasonality and Temporality

Typically, ticks begin searching for a host when the ambient air temperatures are in the range of 4–10 °C. Due to climate change, it is possible that people will engage in outdoor activities earlier in the spring and continue them later into the fall season. This prolonged exposure to tick habitats coupled with an extended period of tick activity increases the likelihood of encountering ticks. On the other hand, during consecutive hot and dry summer days, both outdoor human activity and tick activity are likely to decrease. The heightened risk of human exposure to ticks due to climate change is more closely linked to shorter winters rather than extreme heat events during the summer. [48].

The biodiversity of animal hosts influences the prevalence of pathogens in ticks, which significantly contributes to the expansion of pathogens’ geographic range [49]. One study conducted a Bayesian spatio-temporal binomial regression model to evaluate regional and local temporal trends of *Anaplasma* spp. and *B. burgdorferi* exposure in domestic dogs by analyzing serologic test results for these pathogens. This study found that the regional temporal trends were not static over time, and pathogens increased over time both inside and outside of their historic geographic range. Increased seroprevalence was reported as far as North Carolina and North Dakota for both pathogens. A cluster of counties near West Virginia showed a rise in *B. burgdorferi* seroprevalence. In contrast, another cluster of counties with an elevated prevalence of *Anaplasma* spp. was centered around Pennsylvania and extended into Maine. In the Midwest, clusters within Wisconsin and Illinois saw a decline in seroprevalence for both pathogens, with only Iowa and southwestern Michigan experiencing an increase [50].

Studies have documented numerous tick species that impact humans in the Northeastern US. New Hampshire has one of the highest incidences for LD and anaplasmosis in the US. A study in the state utilized tick surveillance data that reported the seasonal activity of ticks. The study found the highest abundance of *I. scapularis* concentrated in April to June and September to November. Holistically, the study proposed two high suitability areas in the state, specifically southern and central, and recommended that April to August be considered high risk for humans in New Hampshire to encounter *I. scapularis* and *D. variabilis* ticks since this time of the year concentrates the maximum activity of both most prominent tick species [51].

A similar study validated this seasonality by monitoring questing tick activity. The study found that in Wisconsin and Massachusetts, nymphal activity spanned from spring to autumn and peaked in May and June. Concurrently, in these sites, larval activity exhibited a pronounced peak in August, which persisted in autumn [52]. An in-depth analysis conducted in New York examined the seasonality of tick infection rates and found that while the density of ticks is greater in the summer months (June through August), the proportional prevalence of tick infection and the density of infected ticks are significantly higher in the spring (April and May) and fall (September to November) [53]. These differences in the prevalence and density of nymphal ticks are more active during those time periods.

### 3.4. Land Use Factors

In the Eastern US, historical land use patterns have primarily involved a decrease in agriculture and subsequent reforestation. This transition occurred as cropland, pastures, and other cleared lands were abandoned during the 19th and early 20th centuries [17]. Given the strong association between *I. scapularis* and deciduous forests [18], some researchers suggest that the expansion of forests into former agricultural areas and, more recently, into urban areas (such as urban forests or other green spaces) has been and continues to be a significant factor contributing to the emergence of TBDs [54]. In fact, the Environmental Systems Research Institute’s (ESRI’s) projections for landscape changes in Illinois by 2050 indicate an anticipated increase of more than 821,000 acres of farmland across the state, an expansion of over 503,000 acres of urban or impermeable surfaces, and reductions in deciduous forest (743,000 acres), grassland (380,000 acres), and wetlands (39,224 acres) [55]. To enhance the accuracy of future modelling, these forecasts should be integrated to refine the existing static landscape assumptions.

Additionally, habitat fragmentation and heterogeneity result in the conversion of formerly connected forest areas into smaller patches, especially in the Northeastern US. This transformation is expected to have effects on population sizes, movement behaviors, and pathways of disease transmission for small-mammal communities at various ecological levels. A study conducted in Massachusetts that explored the structure of a small-mammal community in terms of mammal abundance and the prevalence of infection with *B. burgdorferi* found that white-footed mice and northern short-tailed shrews in the northeastern part of the state were more abundant in fragmented landscapes at the 500 m and 200 m radii scales, respectively. The study also documented a significantly greater prevalence of *B. burgdorferi* in white-footed mice in interior forests, suggesting differential degrees of exposure in different edge types. Additionally, the study found increased infection prevalence associated with more leaf litter and vegetation cover at fine scales and more wetland edge at the 500 m radius scale [56].

Studies reporting the association between TBDs and landscape factors also reveal the significance of including residential risk assessments for tick exposure. Understanding how ticks infiltrate residential areas is critical for mitigating the abundance of infected ticks through landscape modifications and the implementation of chemical or biological controls. A study conducted in Washington County, Minnesota, reported a collection of *I. scapularis* nymphs from 83% of the residential sites included in the study. The most prevalent forest type found on most residential sites was deciduous forest (67%). Seven sites (30%) primarily featured mixed deciduous and coniferous forests, and one site exclusively contained coniferous trees. Additionally, it was found that there was a four-fold increase in the risk of encountering a host-seeking *I. scapularis* nymph on properties where there were evident signs of deer, a 12-fold increase for properties with the presence of log piles, and an overall 1.5-fold increase for every 10% increase in the amount of forested area on a property [57].

### 3.5. Prevention Strategies

We found a range of prevention strategies implemented across the US. Among the most frequently highlighted control methods were prescribed fires targeting tick populations. Studies have demonstrated that regular prescribed burns can significantly diminish both the abundance of ticks and the incidence of specific TBDs [58]. A study developed a mathematical model that incorporates the effects of prescribed burning in a spatially explicit manner to study the effects of burning on tick populations. One of the main findings of this study was that yearly high-intensity fires were significantly more effective than yearly low-intensity fires. This strategy was also found to slow down the establishment of ticks in new areas [59]. Conversely, another study conducted in West Central Illinois found that the number of ticks collected at all life stages did not differ among burn treatments, which indicates the limited effect of low-intensity burning. The study highlighted the possibility that hosts harboring ticks can immigrate to recently burned areas in search of mast and young vegetation for food and consequently facilitate the reestablishment of tick populations [60].

Numerous strategies have demonstrated promise in reducing the presence of questing ticks or ticks on hosts and in disrupting the transmission, as observed in small-scale field trials [61]. Nevertheless, there has been limited testing of these methods, whether individually or in combination as integrated tick management approaches, in large-scale trials that assess epidemiological outcomes, partly because such studies are expensive [62]. An inexpensive prevention method is utilizing simulation models to generate outcomes that can be readily measured under field conditions. LYMESIM 2.0 is one such mechanistic model simulating the life history of *I. scapularis* and the transmission dynamics of *B. burgdorferi* s.s. Research on the application of this model found that information from this model can be valuable for implementing current tick control strategies and devising and directing new control methods. For example, the model found that the roughly equivalent transmission rates between ticks and both white-footed mice and shrews predict that while a rodent vaccine or treatment could be effective, a more effective approach might involve vaccinating or culling both white-footed mice and shrews [63].

Apart from the various experimental interventions tested to control TBDs, tick surveillance forms a foundation to predict and manage the disease burden in both tick and human populations. A survey conducted across states, counties, and local public health facilities, as well as vector control agencies, revealed a common interest among most jurisdictions in broadening their initiatives. However, various challenges impeded this expansion, including inconsistent funding, restricted infrastructure, a dearth of guidance on optimal practices, insufficient training opportunities for personnel, and limited institutional capability to carry out these functions [64].

## 4. Discussion

In this study, we gathered, reviewed, and synthesized the available published evidence on the complex relationship between climate, land use, the effectiveness of preventive strategies in preventing TBDs, and the incidence of TBDs. We adhered to structured PRISMA guidelines to conduct our review, and we thoroughly searched the most robust literature databases relevant to research on TBDs in the US. Furthermore, we examined the included studies for bias using the ROBINS-I tool, which included relevant domains of bias for our systematic review, and found that our review is minimally susceptible to biases. While we found some inconsistencies across the reviewed manuscripts, our overall results provide a foundation for further research. Our main finding was that more research is needed regarding prevention strategies that consider land use and climate factors. The current body of information available is limited and suggests that localized research focusing on TBD incidence as the primary outcome is needed. However, our review also showed a general support for tailored, localized prevention strategies.

Numerous studies have established the sensitivity of tick lifecycles to climate and weather conditions, considering factors such as their long-life spans, ectothermic physiology, and close interaction with the physical environment [65]. Previous research has shown that temperature and moisture play significant roles in tick mortality, development, and their host-seeking abilities [66]. Both low and high temperatures have been found to decrease the survival and host-seeking activity of *I. scapularis* ticks [67]. Furthermore, cool temperatures prolong tick development and generation periods, leading to greater proportional tick mortality before reproduction. Rainfall and moisture availability also influence host-seeking activity exponentially. Low humidity exposure substantially increases tick mortality, inhibits host-seeking activity, and also determines the height and duration of ticks’ quests above ground [18,21]. Research also established that the average temperature thresholds for the cessation of movement and coordinated movement by *I. scapularis* were 9.8 and 13.9 °C, respectively. However, certain individual nymphs exhibited movement and coordinated movement capabilities at much lower temperatures, namely 4.2 and 6.3 °C, respectively [68]. However, heavy rainfall may also directly impede questing behavior and limit tick–host interactions. Given these ecological relationships, temperature and precipitation are important predictors of these tick species’ latitudinal and altitudinal range limits [25,69]. Additionally, the northward range expansion of *I. scapularis* has been associated with warming temperatures [70]. All Ixodid ticks often modify their questing behavior to remain closer to the moist vegetative surface, avoid desiccating conditions, or return frequently to rehydrate. Both behaviors decrease the probability of obtaining a blood meal and limit survival and reproduction [65].

While research has elucidated the general characteristics and seasonality of LD, information on factors like different host life histories and reservoir competence profiles provides some insight into the disparate LD ecologies, especially on the east and west coasts of the US. In the northeastern region, white-footed mice serve as a significant reservoir for *B. burgdorferi* [71]. The reservoir competence of white-footed mice for *B. burgdorferi* spans approximately 6–8 months, with a decline in infectiousness over time [72]. However, it is crucial to note that white-footed mouse populations exhibit high annual turnover rates; only around 2% of them survive the winter in Connecticut [73]. As a result, the cohort of mice exposed to *B. burgdorferi* in a given year is largely absent the following year. Therefore, it is likely that *B. burgdorferi* persists within the tick population during the winter because most infected mice either die or are no longer infectious between the peak larval activity in late summer and fall and the subsequent peak nymphal activity in the following spring [74]. However, it should be noted that other vertebrate hosts such as shrews, chipmunks, and squirrels can also infect *I. scapularis* ticks, although further investigation is needed to understand the duration and seasonal variations in their reservoir competence [75].

The manner in which humans traverse areas with abundant infectious vectors and reservoir hosts is another contributing factor to the spread of TBDs. For instance, individuals who are active in large residential lots, parks, or agricultural fields that serve as foraging grounds for deer may face an increased risk of contracting LD [76]. While various abiotic, biotic, and behavioral factors influence the spread of TBDs, land cover seems to play a crucial role.

Numerous studies exploring the connection between land cover and TBDs typically employ relatively small, localized study areas. Localized investigations conducted in Maryland [77], the Upper Midwest [78], and the Hudson River Valley of New York [79] have discovered a notable and positive correlation between LD incidence rates and the presence of deciduous forests. However, research conducted in Kent County, Maryland, found that tick abundance exhibits a significant and negative association with forest cover, low-density developed land, and agricultural land [80]. Guerra and colleagues (2002) found that tick population density displays a negative correlation with grasslands and conifer forests [78]. A different study, conducted in Virginia, indicated that the percentage of forest cover is not a significant variable in predicting LD risk [76]. This same study in Virginia [76] supported the findings of another study [77], which found that areas with interspersed herbaceous-forest land exhibit higher incidences of LD. Another study in Virginia [81] discovered that herbaceous cover, particularly scrub, developed-forest edges, and forest-herbaceous edges, has a positive correlation with LD incidence. These discrepancies in findings suggest spatial variability in the abiotic and bioclimatic characteristics (such as moderate climate and moist soils) that influence the densities of both vectors and hosts.

Environmental variation poses challenges when attempting to extend study findings from one location to another [82]. Hence, comprehending the spatial variability in the correlation between land cover and TBDs within broader regions can be valuable in informing localized research and mitigation strategies. By shedding light on potential area and landscape factors that contribute to infection rates, this understanding may help researchers and policymakers develop more effective strategies at the local level.

While it is challenging to control many of the environmental factors, it is important to highlight the importance of efficient and innovative diagnostic procedures to mitigate the impact of these diseases. The onset of the genomics era has stimulated the creation of diagnostic tests utilizing transcriptome analysis, specifically “RNA-Seq,” to examine the human host response [83]. Categorization through gene expression profiling has proven beneficial in recognizing different infections, such as Staphylococcal bacteremia [84], distinguishing between active and latent tuberculosis [85] and identifying influenza [86] and COVID-19 [87]. Transcriptome profiling of peripheral blood mononuclear cells (PBMCs) [88] or skin lesions characteristic of early LD [89] has revealed significant inflammatory responses, primarily dominated by interferon signaling. Analyzing RNA-Seq data through machine learning (ML) has been employed in the classification of cancer [90], although, as of now, it has not been utilized for the diagnosis of infectious diseases. An investigation employing a 31-gene classifier panel for LD showcased its ability to accurately recognize patients with early symptoms from blood samples, occurring within weeks of a tick bite. This panel demonstrated efficacy in identifying cases even before conventional laboratory tests yielded positive results. In the future, the LD Classifier (LDC) holds promise as a clinically useful diagnostic tool for LD, enabling earlier detection of the illness and facilitating more prompt and effective treatment [91].

### 4.1. One Health

A One Health approach recognizes the interconnectedness of humans, animals, and the environment and integrates the work of multiple sectors to address problems together instead of separately. Approximately 60% of emerging infectious diseases reported globally originate from animals, while human activities and burdened environments have created opportunities for disease spread [92]. Even minor shifts in the interlinked relationship between the three components can increase the risk of novel human and animal disease development. A One Health framework can be applied to a range of human and animal diseases, utilizing collaboration across sectors to form novel disease control methods. There is a considerable focus on One Health and TBDs since tick bites can transmit pathogens to humans, many TBDs have animal reservoirs, and the environment has an influence on ticks and the pathogens they carry. With environmental negligence and the recent pandemic, there is a current, increased need for integration and an emphasis on connected health. One Health is a relatively new scientific focus that began in 2003, and critical gaps remain in the approach, including the identification of best practices, model surveillance systems, and methods for identifying and reducing the risk of VBDs. A comprehensive approach to TBDs is needed to fill these gaps with teams comprised of various fields and at local, regional, and national levels [92].

### 4.2. Prevention Strategies

In our review, we found articles discussing a number of prevention strategies, including both personal protective measures and ecological preventative measures. Some of the personal protective measures mentioned involved non-medical interventions, such as the use of permethrin or pyrethroids, wearing longer, light-colored clothing when outdoors, and also some medical interventions, such as the prescription of prophylactic antibiotics. Because of a lack of vaccines for TBDs in the US and common applications of standardized community-based tick management strategies, TBD prevention relies heavily on personal protective behaviors during outdoor and peri-domestic activities in settings to suppress ticks or interrupt enzootic pathogen transmission [93]. Personal protective behaviors encompass precautionary measures that minimize the chances of encountering ticks (such as avoiding high-risk environments and employing repellents), as well as actions that diminish the likelihood of contracting LD following exposure to ticks (such as conducting thorough tick checks and cleansing oneself through showering after outdoor activities, thereby eliminating potentially tick-infested clothing and enhancing the ability to detect ticks) [94]. In a study that used a smartphone application as a novel survey tool (the Tick App), the four most commonly reported personal protective behaviors were ‘Check myself for ticks’ (the most common behavior), ‘Tick repellent (e.g., DEET, picaridin)’, and ‘Wear protective clothing (e.g., light colored, long-sleeved, tucking pants in socks, boots, not including permethrin-treated clothing)’ [33].

Although personal protective measures are often recommended in areas where TBDs are endemic, there is limited evidence regarding their effectiveness. In a randomized controlled trial involving 82 outdoor workers in Rhode Island and southern Massachusetts, long-lasting permethrin impregnation (LLPI) clothing demonstrated a 58% effectiveness in protecting against tick bites over a period of 2 years when compared to untreated clothing [95]. Notably, in the first year of the study, LLPI-treated clothing significantly reduced tick bites by 65%, even when participants in both groups employed other preventive measures against tick bites. However, there was a decrease in the protective effectiveness of LLPI clothing against tick bites from the first to the second year, with a 15-percentage point reduction resulting in a 50% decrease in tick bites during the second year [95]. While a decline in protective effectiveness against tick bites over time was anticipated and has been observed in previous studies [96], the extent of the observed reduction was smaller in this study compared to a previous investigation involving North Carolina outdoor workers [96]. However, it should be noted that the 65% protective effectiveness against tick bites achieved in the first year of this study is lower than the 80% effectiveness observed during the initial year of wearing an LLPI uniform in the study conducted by Vaughn and colleagues [96].

Antibiotic prophylaxis for LD has a history of inconsistent and uncertain results. To counteract that, a systematic review comparing different forms of antibiotic treatments was conducted that revealed that patients who received a single-dose (200 mg) doxycycline course were shown to be less likely to develop LD than those given a placebo (RR, 0.29 (95% CI: 0.14–0.60)), but there was no evidence of the effectiveness of a 10-day course and topical antibiotics course (RR, 0.28 (95%CI: 0.05–1.67) and 0.73 (95%CI: 0.25–2.08)), respectively [97]. Across multiple studies, it was holistically found that no specific personal protection measure stood out as consistently or significantly effective, indicating that there is currently no definitive and universally effective method for primary prevention of diseases transmitted by Ixodes ticks in the US. Commonly advised practices like tick checks, repellent application, and wearing protective clothing yielded varied outcomes across different studies [98].

Concerning ecological preventative interventions, one that is shown to reduce the prevalence of ticks in an area is regularly timed prescribed fires. A recent study exploring the effects of prescribed fires on tick spread and propagation in a spatial setting demonstrated that while ticks can recover relatively quickly following a burn, yearly, high-intensity prescribed burns can reduce the prevalence of ticks in and around that burned area [59]. It was also observed that frequent burning can slow down the establishment of ticks considerably. Another randomized placebo-controlled study tested whether two environmentally safe interventions affected the risk for and incidence of TBDs in humans and pets [99]. The first treatment of interest was a baited box called the Tick Control System (TCS) that brushes the acaricide fipronil onto ticks, and the second treatment was Met52 fungal spray. The utilization of active TCS boxes in residential areas resulted in over a 50% reduction in the number of nymphal ticks actively searching for hosts and approximately a 50% decrease in the presence of ticks on rodents in comparison to placebo controls; however, the application of an active Met52 spray did not show any significant impact on the abundance of either questing ticks or ticks attached to hosts when compared to placebo controls. Additionally, a similar randomized community trial in New York evaluated the same two interventions and their effects on the prevalence of three different pathogens (*B. burgdorferi*, *A. phagocytophilum*, and *Babesia microti*) in black-legged ticks. This study found no significant reduction in prevalence with either intervention when compared to the placebo intervention, but did find cumulative reductions in the prevalence of *B. burgdorferi* with Met52 use [100]. Lastly, a study conducted by the Connecticut Department of Public Health revealed that neither inspecting and removing ticks from one’s body nor using acaricides on personal property were effective strategies for preventing LD [77]. However, it is important to note that this study had certain limitations. The use of these protective measures was based on participant self-reporting, which means that their intervention implementation and effectiveness could not be confirmed or quantified.

### 4.3. Surveillance Strategies

We found that the current public health surveillance strategies for TBDs are varied, reflecting the complexity of monitoring and managing these diseases. Thirteen of our eighty-four (15%) included papers discuss relevant surveillance topics; however, many of the documented efforts were not routine, which is a key component of the definition of public health surveillance [101]. Four of these papers discuss routine tick surveillance, while the others focus on various other data sources, such as environmental surveillance or non-routine tick surveys.

One of the papers included, Mader et al. (2021) [64], was a national survey of tick surveillance programs in the US in 2020, and they surveyed 140 VBD professionals at state and local levels. They found that not all the correspondents had tick surveillance programs in their area; in fact, while nearly all collected information on ticks, only two thirds were engaged in passive surveillance, and less than half of the respondents conducted active surveillance. Only 12% of respondents indicated that their jurisdiction directly conducts or otherwise provides funding for tick control or TBD community interventions. Additionally, most of the active surveillance efforts in the US were found in the Northeast, despite the wide geographic range associated with TBDs. Additionally, our review and the literature included have established a need for the surveillance of reservoir hosts, of which only six correspondents reported assessing reservoirs for pathogen prevalence. Overall, this suggests a limited ability, funding, and capacity to conduct adequate surveillance for TBDs.

Marx (2021) [102] demonstrated an innovative approach by leveraging emergency department visits from the CDC’s National Surveillance System Program, which is the US’s only national syndromic surveillance system for tick bites. However, this only captures patients that seek care and highlights the need to synthesize data across sources to provide a more robust general surveillance strategy for TBDs [103].

Innovative public health surveillance approaches are already underway in some jurisdictions. One study engaged citizen scientists in the active surveillance of host-seeking ticks, where public health experts trained volunteers to actively collect ticks on their woodland properties in Maine. This approach demonstrated the feasibility of using citizen science to actively monitor TBD vectors, a cost-effective strategy replicable elsewhere [104]. Another example of such an effort involves multimodal databases for surveillance, overlaying patient survey data on tick bite encounters and reports of tick-borne diseases with data from other sources, such as canine serological reports. This approach offered county-level information regarding tick-borne disease risk and demonstrated the potential benefit of linking multiple data sources to augment the utility of surveillance systems [105].

The potential increase in the geographic range of ticks influenced by climate and land use factors that we have found in our review underscores the need for enhanced public health surveillance, which suggests the need for the ongoing development of new surveillance strategies. Additionally, based on the critical importance of reservoirs that we have outlined in earlier sections, public health surveillance focusing on reservoirs and intermediary hosts in the TBD cycle may offer vital insights into the transmission dynamics of these diseases, and we did not find evidence of robust reservoir surveillance in the US. A comprehensive and adaptive surveillance strategy is needed to address the evolving landscape of TBDs effectively. This strategy should blend passive and active routine surveillance methods, integrate healthcare system data, and extend beyond only vector monitoring to include reservoir hosts. As an additional cost-effective and community-engaging approach, involving residents by encouraging them to report local tick nuisances can complement more traditional surveillance approaches. Such an integrated approach would enhance public health systems’ capacity to anticipate, identify, and respond to TBD outbreaks in a timely and effective manner.

### 4.4. Limitations

In the papers included in this review, we found evidence for the impacts of climate and land use changes on the distribution and presence of TBDs in the US, combined with overall challenges in setting up and implementing TBD prevention strategies. Generally, the evidence was not strong enough to generate firm conclusions, which could possibly be the result of several limitations.

Firstly, since our study only included published papers and papers written in English, there is the potential for selection bias. Grey literature can constitute an important part of the evidence collected in a literature review, but we did not find any reliable sources from within the US for grey literature. Because TBDs are highly prevalent in other parts of the world, it is possible that we failed to include important background literature written in languages other than English. However, we believe that this limitation did not have a major impact on bias in our screened papers, as most of the publications related directly to the US are likely to have been published in English.

Secondly, by focusing entirely on the US, we eliminated evidence collected from other countries and regions with a high prevalence of TBDs, for instance, many parts of Europe and Russia [106,107]. The relationship between TBDs and climate or land use changes has been widely studied in these areas, while we did not find a similar quantity of evidence published for the US. In addition, the papers we included only covered about 30% of the 50 US states, even though both the pathogens and the ticks transmitting them are known to be distributed in almost every state [108]. The third limitation is a potential additional form of selection bias which could have impacted our results through the way the identified papers were screened and selected. By involving no less than three reviewers in this process, we have significantly reduced the risk of this bias; however, it should still be considered a limitation to the results presented in this paper.

Another important limitation is the fact that only papers deemed relevant or showing ‘positive’ results are submitted for publication and ultimately published. While this remained out of our control, we acknowledge the possible impact of this bias. Furthermore, although we did not find any reliable sources for this, the inclusion of grey literature, such as government-commissioned papers or reports and local state health department reports, could have provided additional perspectives to our findings.

Using the ROBINs-I tool for the risk of bias assessment, we found that most of the studies included in our review had a low risk of bias in critical areas such as confounding, suggesting that the study findings are reliable and free from significant systemic errors. However, we noted a moderate risk of selection bias in a few studies, which warrants a cautious interpretation of their results. No studies exhibited a high risk of bias for any bias domain, and all studies were included in the narrative synthesis.

Overall, the limitations potentially affecting this study are consistent among most literature reviews, and we therefore consider them to have had a negligible impact on our results.

## 5. Conclusions

With the ongoing changes in environmental conditions worldwide, the repercussions for humans and animals and the resultant emergence of zoonotic diseases are becoming increasingly evident. These emerging trends emphasize the need to develop locally tailored warning signs and interventions beyond generalized information. In this paper, we aimed to shed light on key factors that significantly influence the occurrence and transmission of TBDs. Furthermore, we emphasize the need for more consistent scientific evidence and stress the importance of conducting locally consistent research aligned with specific environmental conditions, habitats, behavioral practices, beliefs, and the prevalence of vectors and hosts.

## Data Availability

The data presented in this study are available upon request from the corresponding author.

## Supplementary Materials

The following supporting information can be downloaded at https://www.mdpi.com/article/10.3390/microorganisms12010050/s1: Table S1: Overview of measured or estimated impacts of determinants on TBDs [7,9,18,27,28,40,43,44,46,47,48,50,51,52,53,56,57,59,60,63,64,102,103,105,109,110,111,112,113,114,115,116,117,118,119,120,121,122,123,124,125,126,127,128,129,130,131,132,133,134,135,136,137,138,139,140,141,142,143,144,145,146,147,148,149,150,151,152,153,154,155,156,157,158,159,160,161,162,163,164,165,166,167].
