# Supplementary material for: Assessing the Influence of Climate Change and Environmental Factors on the Top Tick-Borne Diseases in the United States: A Systematic Review
Source: Microorganisms. 2023 Dec 27;12(1):50. doi: 10.3390/microorganisms12010050 (PMC10821204; doi:10.3390/microorganisms12010050)
Supplement: Supplementary file 1 [file microorganisms-12-00050-s001.zip › microorganisms-2761285-supplementary.pdf]

Table S1: Overview of measured or estimated impacts of determinants on TBDs.

Author	Study title	Study area	Study aim	Study design	Study period	Outcomes
Gilliam 2018 [60]	The Influence of Prescribed Fire, Habitat, and Weather on Amblyomma americanum (Ixodida: Ixodidae) in West-Central Illinois, USA	West-Central Illinois, USA	To (1) examine the effect of spring burning and landscape position on tick abundance, (2) identify habitat variables that are likely to affect tick abundance, and (3) determine which weather variables affect tick collection and if these variables are specific to life stage.	Tick surveillance	2015-2016	Results from this study serve as a baseline for the effects of low intensity burns, habitat, and weather patterns on tick presence in a midwestern USA oak woodland community.
Roome 2018 [53]	Lyme Disease Transmission Risk: Seasonal Variation in the Built Environment	United States	To determine the spatial distribution of I. scapularis ticks and the prevalence of the Lyme disease pathogen (B. burgdorferi) to assess the risk of infection during all seasons of the year, especially in built environments with fragmented landscapes.	Cohort study	2012-2014	Tick infection rate is proportionally higher during the fall and spring than summer (30.0-54.7% in fall and 36.8-65.6% in spring vs. 20.0-28.2% in summer).
Dumic 2018 [27]	Ticking Bomb: The Impact of Climate Change on the Incidence of Lyme Disease	United States	To examine the relationship between climatic variables and the reported incidence of LD in 15 states that contribute to more than 95% of reported cases within the Unites States.	Cohort study	2000-2016	Given the increase of 8.6 cases of LD per 100,000 population per county-year associated with a 2°C increase in temperature and the average population for a county-year in the sample of 149,606 persons, they have predicted an increase in the number of LD cases by approximately 6,040 per year in the counties in our sample.
Hahn 2018 [57]	Evaluating acarological risk for exposure to Ixodes scapularis and Ixodes scapularis-borne pathogens in recreational and residential settings in Washington County, Minnesota	Minnesota, USA	To compare tick densities across land use types and to identify environmental factors that might impact nymphal density.	Prevalence study	2016-2016	Residences with the highest densities of nymphs were more likely to have a higher percentage of forest cover, log piles, and signs of deer on their property. In recreational areas, they found the highest nymphal densities both in the wooded areas next to trails as well as on mowed trails.
Linske 2018 [102]	Ixodes scapularis (Acari: Ixodidae) Reservoir Host Diversity and Abundance Impacts on Dilution of Borrelia burgdorferi (Spirochaetales: Spirochaetaceae) in Residential and Woodland Habitats in Connecticut, United States	Connecticut, USA	To test the hypothesis that there would be higher diversity of host species resulting in lower prevalence of B. burgdorferi in white-footed mice (Peromyscus leucopus Rafinesque) (Rodentia: Cricetidae) in forested residential areas.	Surveillance study	2015-2016	Using camera and live small mammal trapping techniques, authors determined there was a greater richness of reservoir host species, significantly higher encounters with hosts, and significantly lower B. burgdorferi host-infection in residential areas as compared to large, intact forested stands.
Sonenshine 2018 [7]	Range Expansion of Tick Disease Vectors in North America: Implications for Spread of	United States	To conduct an in-depth examination of the geographic range expansion and some of the factors influencing the progress of four tick species in North	Narrative review	2018-2018	In addition to their expansion northward, they are also moving westward into drier areas of the North American continent previously considered

	Tick-Borne Disease		America, D. variabilis, A. americanum, A. maculatum and I. scapularis.			inhospitable for their establishment.
Keesing 2018 [109]	The Tick Project: Testing Environmental Methods of Preventing Tick-borne Diseases	New York, USA	To test whether environmental interventions could prevent cases of tick-borne diseases by establishing the Tick Project.	Randomised controlled trial	2016-2020	The authors expect that a 4-year, randomized, placebo-controlled, double-blind design, with two tick-control methods and a relatively high level of replication, conducted at the level of residential neighborhoods, will answer the question of whether an intensive community-based strategy can prevent tick-borne infections.
Herrin 2018 [110]	Canine and human infection with Borrelia burgdorferi in the New York City metropolitan area	New York, New Jersey, Connecticut, and Pennsylvania, USA	To analyze social and environmental factors that may contribute to the risk of B. burgdorferi infection in dogs and compare the estimated canine infection risk to human case reports in the same region.	Cohort study	2001-2010	Canine percent positive tests correlated with population-adjusted human case reports, as well as population density, development intensity, temperature, normalized difference vegetation index, and habitat type.
Johnson 2018 [111]	An Acarological Risk Model Predicting the Density and Distribution of Host-Seeking Ixodes scapularis Nymphs in Minnesota.	Minnesota, USA	To conduct an extensive field survey of host-seeking I. scapularis nymphs and develop a subcounty (30 _ 30 m) resolution model of the DON (density of host-seeking I. scapularis nymphs) to better inform estimates of scatological risk of human exposure to I. scapularis.	Prevalence study	2015-2015	There was substantial heterogeneity observed in predicted DON across the state at the county scale; however, counties classified as high risk for I. scapularis borne diseases and counties with known established populations of I. scapularis had the highest proportion of the county predicted as suitable for host-seeking nymphs(0.13 nymphs/100 m^2).
Eisen 2018 [46]	Modeling Climate Suitability of the Western Blacklegged Tick in California	California	To ensemble species distribution modeling to identify suitable climatic conditions for the tick and restrict the results to land cover classes where the ticks are typically encountered (i.e., forest, grass, scrub-shrub, riparian).	Cross sectional study	1980-2014	All models predict suitable habitat (i.e., forest, grass, scrub-shrub or riparian land cover classes with suitable climatic conditions) for I. pacificus in the coastal regions from Humboldt County south-ward and include suitable habitat in the western foothills of the Sierra Nevada.
McClure 2018 [112]	Reconciling the Entomological Hazard and Disease Risk in the Lyme Disease System	New York, USA	To investigate LD risk indices for artificial landscapes at different forest cover percentages and levels of fragmentation, examining the relationship of a risk index dependent on a patch-level metric-patch size to aggregate landscape statistics.	Ecological modeling	2000-2015	In simulated landscapes, authors find that the model predicts a unimodal relationship between LD incidence and forest cover, mean patch size, and mean minimum distance (a measure of isolation), and a protective effect for percolation probability (a measure of connectivity).
Ogden 2018 [52]	Evidence for geographic variation in life-cycle processes affecting phenology of the Lyme disease vector *Ixodes scapularis*		To analyze data on questing tick activity and/or tick activity determined by tick counts on captured animal hosts to elucidate the observed seasonal activity patterns at	Ecological study	2010-2012	Temperature-independent diapause mechanisms explain some key observed variations in I. scapularis seasonality and are responsible in part for geographic variations in I.

	(Acari: Ixodidae) in the United States.		multiple sites in the United States.			scapularis seasonality in the United States.
MacDonald 2018 [113]	Abiotic and habitat drivers of tick vector abundance, diversity, phenology, and human encounter risk in southern California	California, USA	To characterize patterns of seasonal activity of the more common vector species and understand seasonality in risk of vector exposure, while specifically focusing on human encounter risk using standardized tick drags.	Tick collection study	2013-2015	I. pacificus may be expected to experience reductions in geographic distribution and seasonal activity under projected land cover and climate change in coastal southern California, while D. occidentalis may experience more limited effects.
Sharareh 2019 [114]	Risk Factors of Lyme Disease: An Intersection of Environmental Ecology and Systems Science	New York, USA	To evaluate the LD risk and the risk of exposure to infected ticks associated with vegetation, walkway types, human risk factors, and vector and reservoir data on a mid-sized university campus in upstate New York, where LD is endemic.	Multinomial logistic regression modelling	2014-2014	The model classifies the walkways into high-risk and low-risk categories with 90% accuracy, in which the understory, human risk, and number of rodents are significant indicators.
Bouchard 2019 [48]	Increased risk of tick-borne diseases with climate and environmental changes	North America	To summarize the climate and other environmental changes affecting the risk of ticks and tick-borne diseases (TBDs), identify the ticks and TBDs that are occurring or that may spread into Canada and describe the public health and clinical strategies for the management of ticks and TBDs.	Text and opinion	2019-2019	Increased temperature increases the survival and activity period of ticks, increases the range of both reservoir and tick hosts (e.g. mice and deer) and increases the duration of the season when people may be exposed to ticks.
Klarenberg 2019 [115]	Evaluation of NEON Data to Model Spatio-Temporal Tick Dynamics in Florida	Florida, USA	To estimate the abundance of tick nymphs and adult ticks across a Florida NEON locations.	Surveillance study	2014-2018	The study found an increase in tick abundance at this Florida location and was able to explain spatial and temporal variability in abundance and detection. showing the potential of NEON data.
Thapa 2019 [28]	Effects of temperature on bacterial microbiome composition in Ixodes scapularis ticks	United States	To better understand the effects of environmental temperature on the microbial communities of ticks, by investigating the bacterial microbiomes of colony-reared I. scapularis ticks statically incubated in a controlled laboratory setting.	Experimental study	2010-2017	The male ticks incubated at 30 and 37Â°C exhibited significantly different bacterial diversity compared to the initial baseline microbiome, and the change in bacterial diversity was dependent upon duration of exposure.
McClure 2019 [116]	Climate impacts on blacklegged tick host-seeking behavior	Eastern USA	To examine the theoretical impacts of climate on the behavior and fitness of I. scapularis, both for locally adapted (optimal) regional subpopulations and for ticks that have been exposed to new climates	Cohort study	2014-2014	The optimal predicted questing strategy changed non-linearly as leaf litter and air water vapor activity crossed the ticks critical equilibrium activity (CEA)- a physiological threshold below which the tick was unable to maintain a steady state water balance.

Lin 2019 [117]	The effects of multiyear and seasonal weather factors on incidence of Lyme disease and its vector in New York State	New York State, USA	To examine the associations of Lyme disease and its vectors with weather factors, especially multi-year and multi-weather factors related to vector life cycle.	Generalized estimating equation modeling	1991-2006	The results indicate that summer Lyme disease cases and tick encounters may increase by 4-10%, per one day in spring with a minimum temperature range between 40 and 50 Â°F in the year of diagnosis and previous year.
Schubert 2019 [118]	Modeling Lyme Disease Host Animal Habitat Suitability, West Point, New York	New York, USA	To identify probable areas where West Point cadets as well as active duty service members stationed at West Point and their families might contract Lyme disease, this study used Geographic Information System mapping methods and remote sensing data to replicate an established spatial model to identify the likely habitat of a key host animal the white-tailed deer.	Spatial modelling study	2019-2019	Urban population centers with reduced green space, increased density of roads, and continuous vehicular traffic offer reduced food and shelter for white-tailed deer, resulting in lower suitability values.
Salkeld 2019 [119]	Time of year and outdoor recreation affect human exposure to ticks in California, United States	California, United States	To examine the importance of human activity (e.g., recreational activity in natural areas) and time-of-year for tick exposure to three tick species.	Prevalence study	2016-2019	Tick exposure occurred predominantly during recreational use of the outdoors, rather than exposure near the home environment. Tick submissions peaked in May, but human exposure to ticks occurred throughout the year.
Gleim 2019 [120]	Frequent Prescribed Fires Can Reduce Risk of Tick-borne Diseases	Georgia and Florida, USA	To directly investigate the impacts of long-term prescribed burning of ticks on human disease risk	Experimental study	2010-2011	Long-term prescribed fire did not significantly impact pathogen prevalence except that A. americanum from burned habitats had significantly lower prevalence of Rickettsia compared to ticks from control sites.
Thomas 2020 [121]	Environmental Drivers of Questing Activity of Juvenile Black-Legged Ticks (Acari: Ixodidae): Temperature, Desiccation Risk, and Diel Cycles	United States	To disentangle the factors that control questing behavior by comparing questing activity at various times of day and night and microclimatic conditions at 10 locations in a research site in the core of I. scapularis range.	Cohort study	2017-2017	Activity of nymphs increased slightly during dawn and dusk, opposite of larvae, and declined slightly with air temperature and rain, but these patterns were weak and inconsistent among replicate sites.
Ginsberg 2020 [18]	Local abundance of Ixodes scapularis in forests		To evaluate the environmental factors that affect local distribution and abundance of I. scapularis.	Cohort study	2010-2012	Canopy cover was predictive of larval and nymphal numbers in flag/drag samples, but not of numbers on hosts.
Gaff 2020 [63]	LYMESIM 2.0: An Updated Simulation of Blacklegged Tick (Acari: Ixodidae) Population Dynamics and Enzootic Transmission of Borrelia burgdorferi (Spirochaetales: Spirochaetaceae)	New York, Minnesota, and Virginia	To present the updated version of the LYMESIM model which is a mechanistic model that simulates the life-history of I. scapularis and transmission dynamics of B. burgdorferi s.s.	Validation study	2007-2016	Sensitivity analysis highlighted the importance of temperature in host finding for the density of nymphs, the importance of transmission from small mammals to ticks on the density of infected nymphs, and temperature-related tick survival for both density of nymphs and infected nymphs.

Dong 2020 [122]	Comparing the Climatic and Landscape Risk Factors for Lyme Disease Cases in the Upper Midwest and Northeast United States	United States	To explore and compare the climatic and landscape factors that shape the spatial patterns of human Lyme cases in Upper Midwest and Northeast, using the generalized linear mixed models	Cohort study	2012-2016	In the Northeast, the total edge length of low intensity developed area, the edge density of deciduous forests and the percentage of high intensity developed had positive effects on Lyme disease risk; while the percentage of evergreen forests have a negative effect.
Sholty 2020 [123]	Borrelia burgdorferi and Anaplasma phagocytophilum Genospecies in Northern California	California, USA	To detect whether B. burgdorferi s.l. and A. phagocytophilum s.l. genospecies-host associations varied by site, host sex, or species, or temporally, and whether there was significant spatial clustering of particular genospecies at four high-risk sites in California.	Cohort study	2006-2013	Woodrats were significantly more likely to be PCR-positive for B. bissettiae and A. phagocytophilum DU1 genospecies, while A. phagocytophilum s.s. and B. burgdorferi s.s. were significantly associated with sciurids.
El-Sayed 2020 [124]	Climatic changes and their role in emergence and re-emergence of diseases	North America, Europe	To review, the role of climate change in the spread of infectious agents and their vectors.	Narrative review	2020-2020	The disturbance in the balanced ecosystems will not only lead to the emergence of infectious diseases but also has long-term serious direct and indirect damaging effects which threaten the existence of humans, animals and plants.
MacDonald 2020 [47]	Projected climate and land use change alter western blacklegged tick phenology, seasonal host-seeking suitability and human encounter risk in California	California, USA	To investigate how environmental suitability for tick host-seeking changes seasonally, how the magnitude and direction of changing seasonal suitability differs regionally across California, and how land use change shifts human tick-encounter risk across the state.	Species distribution modeling	1980-2015	Under a hotter, drier scenario and more extreme land use change, the duration and extent of seasonal host-seeking activity increases in northern California but declines in the south.
Rau 2020 [125]	Spatio-Temporal Dynamics of Tick-Borne Diseases in North-Central Wisconsin from 2000-2016	North-Central Wisconsin, USA	To test the hypothesis that the risk of non-Lyme tick-borne diseases was not stationary in space or time within a large healthcare population in north and central Wisconsin.	Cohort study	2000-2016	Seasonal trends in laboratory positive cases for all diseases indicated summer months (June-August) had the greatest number of cases, whereas winter months (December-February) had the lowest number of cases.
Hahn 2020 [126]	Establishing a baseline for tick surveillance in Alaska: Tick collection records from 1909-2019	Alaska, USA	To review historical tick records in Alaska and summarize recent tick occurrence records collected through the development of the Alaska Submit-A-Tick Program and through tick drag sampling at sentinel sites in southcentral Alaska.	Narrative review	1909-2019	Almost half of the records (n_=_68, 48 %) of non-native tick species from 2010 to 2019 represented ticks found on a host (usually a dog or a human) that had traveled outside of Alaska in the two weeks prior to collection.
Lefcort 2020 [127]	Behavioral characteristics and endosymbionts of two potential tularemia and Rocky Mountain	Washington State, U.S.A	To measure behavioral and ecological differences between the Dermacentor andersoni and D. variabilis ticks and determine which, if any, Rickettsia and	Experimental study	2019-2019	Authors found that D. andersoni is more resistant to desiccation, but both species share similar questing behaviors such as climbing and attraction to bright light.

	spotted fever tick vectors		Francisella bacteria pathogenic or endosymbiotic - they carried.			
Gettings 2020 [50]	Regional and Local Temporal Trends of Borrelia burgdorferi and Anaplasma spp.Seroprevalence in Domestic Dogs: Contiguous United States 2013-2019	United States	To explore canine seroprevalence of B. burgdorferi and to use contemporary dataset and apply the model to canine Anaplasma spp. seroprevalence to identify increases in canine Anaplasma spp. or B. burgdorferi seroprevalence within the United States.	Bayesian spatio-temporal binomial regression model	2013-2019	A large cluster of counties with increased B. burgdorferi seroprevalence centered around West Virginia, while a similar cluster of counties with increased Anaplasma spp. seroprevalence centered around Pennsylvania and extended well into Maine
Elias 2021 [138]	A Generalized Additive Model Correlating Blacklegged Ticks with White-Tailed Deer Density, Temperature, and Humidity in Maine, USA, 1990-2013.	Maine, USA	To use a generalized additive model to assess linear and nonlinear relationships between I. scapularis nymph abundance and predictors.	Cohort study	1990-2013	Nymph submission rate increased with increasing deer densities and were associated with warmer minimum winter temperatures, earlier degree-day accumulation, and higher relative humidity.
Winter 2021 [166]	Modeling the Sensitivity of Blacklegged Ticks (Ixodes scapularis) to Temperature and Land Cover in the Northeastern United States	Northeast USA	To improve upon a recently developed compartment model of ordinary differential equations that simulates I. scapularis growth, abundance, and infection with Borrelia burgdorferi (Spirochaetales: Spirochaetaceae) by adding land cover effects on host populations, refining the representation of growth stages, and evaluating output against observed data.	Ecological modelling	2011-2011	Consistent with observations, simulations of I. scapularis abundance are sensitive to temperature, with the warmer Storrs climate significantly increasing the number of questing I. scapularis at all growth stages.
Ghosh 2021 [140]	Ixodes spp. from Dogs and Cats in the United States: Diversity, Seasonality, and Prevalence of Borrelia burgdorferi and Anaplasma phagocytophilum		To determine the diversity of Ixodes spp. feeding on dogs and cats throughout the United States, the haplotypes and seasonality of I. scapularis infesting pets in the eastern United States, and the prevalence of infection with pathogens in different regions.	Northeast, Midwest, Southeast, and	2018-2019	Seven different species of Ixodes ticks were recovered from pets in the United States, and the diversity of Ixodesspp. was particularly high in the Southeast and the West.
Diuk-Wasser 2021 [137]	Impact of Land Use Changes and Habitat Fragmentation on the Eco-epidemiology of Tick-Borne Diseases	United States	To examine how land use interacts with abiotic conditions (microclimate) and biotic factors (e.g., host community composition) to influence the enzootic hazard, measured as the density of host-seeking I. scapularis nymphs infected with B. burgdorferi s.s.	Narrative review	2021-2021	The land use legacies of Europe and North America intersect with ecological and sociological factors to construct Lyme disease emergence patterns more broadly.

Couper 2021 [40]	Impact of prior and projected climate change on US Lyme disease incidence	United States	To utilize 18 years of annual, county-level Lyme disease case data to investigate prior effects of climate variation on disease incidence while controlling for other putative drivers.	Cohort study	2000-2017	The interannual variation in Lyme disease incidence is associated with climate variation in all US regions encompassing the range of the primary vector species. The strongest climate-disease association detected was between warming annual temperatures and increasing incidence in the Northeast.
Tufts 2021 [164]	Ixodes scapularis (Acari: Ixodidae) Nymphal Survival and Host-Finding Success in the Eastern United States	Connecticut and Rhode Island, USA	To devise a model for estimating host-finding success from the ratio of tick abundance in two subsequent years, accounting for overwinter survival and possible differences in host associations between nymphs and larvae.	Prevalence study	2015-2016	Assuming equivalent host association between larvae and nymphs, R0 was also estimated to be greater than one at three of four sites, suggesting these conditions allow for the persistence of B. burgdorferi.
Tran 2021 [163]	Spatio-temporal variation in environmental features predicts the distribution and abundance of Ixodes scapularis	New York, USA	To develop spatio-temporal biogeographic models to assess the potential impact of hundreds of environmental features, derived from remotely sensed climate and landscape data, on the occurrence and abundance of I. scapularis nymphs.	Ecological study	2008-2018	Three broad regions associated with higher elevation in mountainous areas are predicted to have a low probability of nymphal tick populations throughout all sampling years including 2018, although the size of the geographic expanse of the broad areas with low probabilities of nymphs is variable among years.
Porter 2021 [155]	Predicting the current and future distribution of the western black-legged tick, Ixodes pacificus, across the Western US using citizen science collections	Western USA	To use MaxEnt species distribution models to predict the current and future distribution of Ixodes pacificus across the Western US through the use of a nationwide citizen science tick collection program.	Ecological niche modeling	2016-2023	Citizen science tick collections and other collection methods, such as mobile smartphone application-based citizen science programs (e.g., The Tick App) and photographic identification of passively collected ticks (e.g., TickSpotters), have the potential to fulfill this need and allow for a variety of questions to be answered.
Ginsberg 2021 [43]	Why Lyme disease is common in the northern US, but rare in the south: The roles of host choice, host-seeking behavior, and tick density	Sites in Wisconsin, Massachusetts, Rhode	To compare tick vertebrate host interactions using standardized sampling methods among 8 sites scattered throughout the eastern US.	Prevalence study	2010-2012	Tick infection prevalence declines north to south largely because of high tick infestation of efficient spirochete reservoir hosts (rodents and shrews) in the north but not in the south.
Small 2021 [158]	Detection of Rickettsia amblyommatis and Ehrlichia chaffeensis in Amblyomma americanum Inhabiting Two Urban Parks in Oklahoma	Oklahoma, USA	To assess the prevalence of tick-borne pathogens in different environments, in two parks in Edmond, Oklahoma.	Prevalence study	2016-2017	Higher case rates of Rocky Mountain spotted fever (RMSF) and SFG rickettiosis (SFGR) than ehrlichiosis in Oklahoma coupled with lower prevalence of Dermacentor variabilis ticks, the primary vector of Rickettsia rickettsii, may be an indication that R. amblyommatis is responsible for some of the RMSF or RMSF-like illnesses reported in Oklahoma.

Meyers 2021 [152]	Epidemiology of Vector-Borne Pathogens Among U.S. Government Working Dogs	United States	To determine the levels of infection and exposure to several vector-borne pathogens among working dogs across the United States that have high outdoor exposure and work and live in close proximity to humans.	Cohort study	2015-2018	Seroprevalence of each pathogen was: B. burgdorferi 0.84%, Ehrlichia spp. 1.3%, Anaplasma spp. 1.5%, Leishmania spp. 2.0%, and T. cruzi 12.2%. Coinfection or co-exposure took place in four (0.84%) dogs.
Backus 2021 [44]	Effect of Temperature on Host Preference in Two Lineages of the Brown Dog Tick, Rhipicephalus sanguineus.	Maricopa, Arizona, USA	The aim of this study was to assess the impact of hot weather on choice between humans and dog hosts among tropical and temperate lineage Rh. sanguineus individuals.	Non-randomized experimental study	2021-2021	Short-term weather patterns and climatic change may both impact the transmission of pathogens transmitted by Rh. sanguineus to humans.
Lantos 2021 [148]	Environmental Correlates of Lyme Disease Emergence in Southwest Virginia, 2005Ð2014	Southwest Virginia, USA	To determine which environmental variables were	Cross sectional study	2005-2014	Higher elevation and higher vegetation density had the greatest effect size on the abundance of Lyme disease.
Hacker 2021 [142]	A Comparison of Questing Substrates and Environmental Factors That Influence Nymphal Ixodes pacificus (Acari: Ixodidae) Abundance and Seasonality in the Sierra Nevada Foothills of California	California, USA	To evaluate the efficiency in collecting Ixodes pacificus nymphs, characterize nymphal seasonality, and identify environmental factors affecting their abundance and infection with B. burgdorferi sl.	Tick sampling	2015-2017	Substrate type, collection year, month, and canopy cover were all significant predictors of nymphal density and prevalence. Logs, rocks, and tree trunks had significantly greater nymphal densities and prevalences than leaf litter.
Marx 2021 [103]	Emergency Department Visits for Tick Bites, United States, January 2017 - December 2019	United States	To use data from CDC's National Syndromic Surveillance Program (NSSP), investigators examined ED tick bite visits during January 2017 -December 2019 by sex, age group, U.S. region, and seasonality.	Syndromic surveillance report	2017-2019	Syndromic surveillance data for tick bites can guide timely, actionable public health messaging such as avoiding tick habitats, wearing repellent consistently when outdoors, and performing regular tick checks during times of increased tick bite risk.
Heaney 2021 [144]	Relations of peri-residential temperature and humidity in tick-life-cycle-relevant time periods with human Lyme disease risk in Pennsylvania, USA	Pennsylvania, USA		Case control study	2006-2014	Exposure-response patterns were observed for higher cumulative same-year temperature, humidity, and hot and dry days (nymph-relevant), and prior year hot and dry days (larva-relevant), with same-year hot and dry days showing the strongest association.
Mader 2021 [64]	A Survey of Tick Surveillance and Control Practices in the United States.	United States	To assess 1) tick surveillance program objectives, 2) pathogen testing methods, 3) tick control practices, 4) data communication strategies, and 5) barriers to program development and operation.	Tick surveillance	2018-2018	Most of the programs currently supporting tick pathogen testing were in the Northeast (70.8%), Upper and Central Midwest (64.3%), and the West (71.4%) regions. The most common pathogens screened for were Rickettsia spp. (Rickettsiales: Rickettsiaceae) and bacterial and viral agents trans-mitted by Ixodes (Acari: Ixodidae) ticks.

Hahn 2021 [143]	Modeling future climate suitability for the western blacklegged tick, Ixodes pacificus, in California with an emphasis on land access and ownership	California, USA	To explore how climate change may alter the geographic distribution of I. pacificus in California, USA, during the 21st century, and assess the spatial overlap among predicted changes in tick habitat suitability, land access, and ownership.	Species distribution modelling	2026-2099	Over a third of the future suitable habitat is on lands currently designated as open access (i.e., publicly available), and by 2100, the amount of these lands that are suitable habitat for I. pacificus is projected to more than double under the most extreme emissions scenario.
Russell 2021 [9]	Epidemiology and Spatial Emergence of Anaplasmosis, New York, USA, 2010â€'2018	New York, USA	To examine human case surveillance and tick surveillance data during 2010-2018 to assess the epidemiology, risk for pathogen exposure, and spatiotemporal emergence patterns of anaplasmosis in NYS	Surveillance and standardized drag surveys	2010-2018	Spatial analysis showed an expanding hot spot of anaplasmosis in the Capital Region, where incidence increased >8-fold.
O'Connor 2021 [154]	A Comparative Spatial and Climate Analysis of Human Granulocytic Anaplasmosis and Human Babesiosis in New York State (2013-2018)	New York State, USA	To use a large-scale spatially weighted regression to comparatively examine the association between the environment of NYS and the rates of HGA and human babesiosis.	Cohort study	2013-2018	Surveillance data of tick-borne pathogens (2007 to 2018) and reported human cases of HGA (n = 4,297) and human babesiosis (n = 2,986) (2013-2018) from the New York State Department of Health (NYSDOH) showed a positive association between the presence/temporal emergence of each pathogen and rates of disease in surrounding areas.
Neupane 2021 [153]	Spatio-temporal modeling for confirmed cases of LD in Virginia	Virginia, USA	To observe ecological and environmental covariates and construct a predictive model for the disease spread over space and time, including spatial and temporal random effects.	Cohort study	2001-2016	Among the various ecological predictors, the North-South (V component) of winds and relative humidity significantly contributed to predicting the Lyme cases.
Ginsberg 2021 [145]	Potential Effects of Climate Change on Tick-borne Diseases in Rhode Island	Rhode Island, USA	To identify the major tick-borne diseases that currently occur in Rhode Island, describe the underlying transmission dynamics, and consider the likely effects of climate change.	Narrative review	2010-2019	Human cases of several tick-associated diseases in Rhode Island, Lyme disease chief among them, but also anaplasmosis, babesiosis, ehrlichiosis, and alpha-gal syndrome, are likely to show northward range expansion.
Schimpf 2021 [156]	Responses of ticks to immersion in hot bathing water: Effect of surface type, water temperature, and soap on tick motor control	United States	To investigate the response of adult and nymphal ticks to simulations of immersion in hot bath water to infer whether several physical factors of bathing water could induce non-attached ticks to lose contact with skin.	Experimental study	2021-2021	Among the adults tested, the female and male held in 41ËšC plain water for 20 min did not lose leg movement by the end of that exposure. All other ticks tested were unresponsive to prodding when first removed from their exposure tube.
Lippi 2021 [149]	Trends and Opportunities in Tick-Borne Disease Geography	Global	To systematically review studies that map tick-borne diseases using data on vectors, pathogens, and hosts (including wildlife, livestock, and human cases)	Systematic review	1995-2020	Authors find a tremendous diversity of methods used to map tick-borne disease, but also find major gaps: data on the enzootic cycle of tick-borne pathogens is severely underutilized, and mapping efforts are mostly limited to Europe and North America.

Alkishe 2021 [129]	Likely Geographic Distributional Shifts among Medically Important Tick Species and Tick-Associated Diseases under Climate Change in North America: A Review	United States	To assess whether and how species distributions will likely shift in coming decades in response to climate change.	Narrative review	2021-2021	The eastern US appears to hold more medically important tick species, which may make it present higher risk of tick-associated disease transmission than in the central and western US.
Sundheim 2021 [159]	Seasonality of Acute Lyme Disease in Children	United States	To explore the seasonality of Lyme disease overall and by clinical presentation in a multi-center cohort of children with acute Lyme disease.	Cohort study	2001-2002	While most children with early or early-disseminated Lyme disease presented during the summer months, children with Lyme arthritis presented throughout the year.
Volk 2022 [165]	Microclimate conditions alter Ixodes scapularis (Acari: Ixodidae) overwinter survival across climate gradients in Maine, United States	Maine, United States	To explore whether winter climate currently limits the distribution of blacklegged ticks and the pathogens they transmit in Maine, U.S.A., by contributing to overwinter mortality of nymphs.	Experimental study	2018-2020	The results suggest that overwinter survival is not the sole constraint on the blacklegged tick distribution even under extremely cold ambient conditions and additional mechanisms may limit the continued northward expansion of ticks.
Bacon 2022 [131]	Effects of Climate on the Variation in Abundance of Three Tick Species in Illinois	United States	To determine if climate factors explain existing differences in abundance of the three aforementioned tick species between two climatically different regions of Illinois (Central and Southern), and if climate variables impact each species differently.	Retrospective study (tick drags data)	2018-2019	Results suggested that the maximum average temperature and total precipitation are associated with differential impact on species abundance and that this difference varied by region.
Witmer 2022 [167]	Modeling Geographic Uncertainty in Current and Future Habitat for Potential Populations of Ixodes pacificus (Acari: Ixodidae) in Alaska	Alaska, USA	To use habitat suitability models for I. pacificus in the contiguous United States to estimate potentially suitable habitat in Alaska based on present-day climate and future climate scenarios.	Forecasting model	1980-2100	The present-day habitat suitability maps suggest that the climate and land cover in Southeast Alaska and portions of Southcentral Alaska could support the establishment of I. pacificus populations.
Fulk 2022 [59]	Exploring the Effects of Prescribed Fire on Tick Spread and Propagation in a Spatial Setting	United States	To develop a mathematical model that incorporates the effect of prescribed burning in a spatially explicit manner in order to investigate both short-term and long-term effects of burning on tick populations in different scenario.	Mathematical modelling	2022-2022	Results indicate that while ticks can recover relatively quickly following a burn, yearly, high-intensity prescribed burns can reduce the prevalence of ticks in and around the area that is burned.
Bajwa 2022 [132]	The Gulf Coast Tick, Amblyomma maculatum (Ixodida: Ixodidae), and Spotted Fever Group Rickettsia in the Highly Urbanized Northeastern United States	United States	To report the multi-year collection of the Gulf Coast tick, Amblyomma maculatum Koch (Acaridae: Ixodida: Ixodidae) in Staten Island, New York City (NYC) as well as their detection in Brooklyn, NYC, and in Atlantic and Cumberland counties in southern New Jersey, USA.	Environmental Surveillance	2018-2018	Freshkills Park, appears to provide many of the biotic and abiotic features that are key determinants of the survival and establishment of A. maculatum in an area. This includes large grasslands in mid-to-late successional stages and abundant populations of small mammals and birds as well as white-tailed deer, a major host of the adults.

Jordan 2022 [146]	Relevance of Spatial and Temporal Trends in Nymphal Tick Density and Infection Prevalence for Public Health and Surveillance Practice in Long-Term Endemic Areas: A Case Study in Monmouth County, NJ	Monmouth County, New Jersey, USA	To report high levels of spatiotemporal variability in nymphal density and infection prevalence in xodes scapularis Say (Acari: Ixodidae) and Amblyomma americanum L. (Acari: Ixodidae), limiting the granularity with which human risk can be predicted from acarological data.	Surveillance study	2017-2021	The authors saw a considerable spatiotemporal variability in the nymphal density of both tick species, among and within sites, density estimates were relatively consistent among regions over the 5-yr period, with Inner Coastal Plain sites yielding the lowest numbers of I. scapularis nymphs and Piedmont sites the highest numbers.
Mason 2022 [56]	Multi-scale analysis of habitat fragmentation on small-mammal abundance and tick-borne pathogen infection prevalence in Essex County, MA	Massachusetts, USA	To determine the structure of a small-mammal community in terms of mammal abundance and infection prevalence of Borrelia burgdorferi sensu stricto (s.s.), Anaplasma phagocytophilum, and Babesia microti within a fragmented landscape in Essex County, Massachusetts, USA.	Generalized linear modeling	2012-2013	White-footed mouse abundance was negatively associated with amount of forested area within a 500-m radius, whereas northern short-tailed shrew abundance demonstrated a positive relationship with fragmentation indices at the 200-m radius.
Flenniken 2022 [139]	Environmental Drivers of Gulf Coast Tick (Acari:Ixodidae) Range Expansion in the United States	Illinois, USA	To use an ecological niche modeling approach and combine new occurrence records with high-resolution climate and land cover data to investigate environmental drivers of the current distribution of A. maculatum in the United States.	Cohort study	2020-2020	The presence of A. maculatum was associated with open habitat with minimal canopy cover. The model predicts large areas beyond the current distribution of A. maculatum to be environmentally suitable, suggesting the possibility of future northward and westward range expansion.
Couret 2022 [135]	The environment, the tick, and the pathogen-It is an ensemble	United States	To highlight (With a focus on B. burgdorferi) the interplay of abiotic factors such as temperature and humidity as well as biotic factors such as environmental microbiota that ticks are exposed to during their on-and off-host phases on tick, and infection prevalence	Narrative review	2022-2022	The impact of humidity on tick survival and host-seeking behavior is profound, and I. scapularis may be more sensitive to perturbations in humidity than temperature.
Alkishe 2022 [128]	Climate change influences on the geographic distributional potential of the spotted fever vectors Amblyomma maculatum and Dermacentor andersoni	United States	To use ecological niche modeling (ENM) to assess the potential geographic distributions of Amblyomma maculatum (Gulf Coast tick), and Dermacentor andersoni (Rocky Mountain wood tick) in North America under current condition and then transfer those models to the future under different future climate scenarios with special interest in highlighting new potential expansion areas	Modelling study	2022-2022	Current model predictions for A. maculatum showed suitable areas across the southern and Midwest US, and east coast, western and southern Mexico. For D. andersoni, models showed suitable areas across northwestern US. New potential for range expansions was anticipated for both tick species northward in response to climate change, extending across the Midwest and New England for A. maculatum, and still farther north into Canada for D. andersoni

Backus 2022 [130]	Tick-borne pathogens detected in sheltered dogs during an epidemic of Rocky Mountain spotted fever, a One Health challenge	United States	To assess exposure to and infection with 3 pathogens (Rickettsia rickettsii, Anaplasma platys, and Ehrlichia canis) vectored by brown dog ticks (Rhipicephalus sanguineus) in sheltered dogs at the western US-Mexico border.	Cross sectional study	2021-2022	While infection and exposure were highest in sheltered dogs in the southern locations, dogs in all locations demonstrated exposure to all pathogens, demonstrating the potential for emergence and spread of zoonotic pathogens with significant public health consequences in southern California and northern Baja California.
Semenza 2022 [157]	Climate Change and Cascading Risks from Infectious Disease	Global	To examine cascading risk pathways from climate change for vector-, water-, food-, and air-borne infectious diseases in a global context.	Systematic review	2017-2022	As for tick-borne diseases, climate change is projected to continue to contribute to the spread of Lyme disease and tick-borne encephalitis, particularly in North America and Europe.
Cull 2022 [136]	Monitoring Trends in Distribution and Seasonality of Medically Important Ticks in North America Using Online Crowdsourced Records from iNaturalist	United States	To examine tick observations from the online image-based biological recording platform iNaturalist to evaluate its use as an effective tool for monitoring the distributions of A. americanum, A. maculatum, I. scapularis, and Dermacentor in the United States and Canada.	Cross sectional study	2017-2021	County-level iNaturalist tick occurrence data showed good agreement with other data sources in documented areas of I. scapularis and A. americanum establishment and highlighted numerous previously unreported counties with iNaturalist observations of these species.
Tiffin 2022 [161]	Tick Control in a Connected World: Challenges, Solutions, and Public Policy from a United States Border Perspective	United States	To (1) identify the major modern challenges to tick control and (2) develop best management strategies for tick control given these challenges.	Text and opinion	2022-2022	International inter-agency collaborations along the U.S-Mexico border have been critical in control and mitigation of cattle fever ticks (Rhipicephalus spp.) and highlight the need for continued collaboration and research into integrated tick management strategies
Tran 2022 [162]	Predicting spatio-temporal population patterns of Borrelia burgdorferi, the Lyme disease pathogen	New York, USA	To develop spatio-temporal biogeographic models to investigate the potential effect of environmental features on nymphal infection prevalence (NIP).	Ecological study	2008-2019	The model indicates that NIP is negatively associated with landscape features representing habitat disturbance like recent wildfire, patch connectivity, human population size and critical environment designations.
Tardy 2023 [160]	Mechanistic movement models to predict geographic range expansions of ticks and tick-borne pathogens: Case studies with Ixodes scapularis and Amblyomma americanum in eastern North America	United States	To gain insight into the sensitivity of infected tick spread patterns to temperature increases, by conducting simulation experiments.	Mechanistic movement modelling	2021-2021	The results indicate that the northward invasion process of lone star ticks is primarily driven by local dispersal of resident terrestrial hosts, whereas that of blacklegged ticks is governed by long-distance migratory bird dispersal.
Fernandez-Ruiz 2023 [51]	Passive collection of ticks in New Hampshire reveals species-specific patterns of distribution and activity	New Hampshire , USA	To summarize the ticks collected from humans, pets, and other domestic animals, pinpointing conclusions about the distribution and activity period(s) of the ticks, the most common situations	Cohort study	2018-2021	The combination of active surveys, community science, and predictive mapping may be a compelling source of information, increasing the knowledge about the distribution of I. scapularis

			of risk, the pathogens carried.			and D. variabilis; thus, improving tick bite and tick-borne disease prevention campaigns.
Burtis 2023 [134]	Identifying suitable habitat for Ixodes scapularis (Acari: Ixodidae) infected with Anaplasma phagocytophilum (Rickettsiales: Anaplasmataceae), Babesia microti (Piroplasmida: Babesiidae), and Borrelia miyamotoi (Spirochaetales: Spirochaetaceae) to guide surveillance efforts in the eastern United States	United States	To estimate ranges of suitable habitat for Anaplasma phagocytophilum, Babesia microti, and Borrelia miyamotoi in the eastern United States based on existing county-level surveillance records.	Modeling ensemble	2021-2021	Among counties where the pathogen of interest had not been reported, they identified 431, 275, and 539 counties classified as highly suitable for A.phagocytophilum, Ba. microti, and Bo. miyamotoi, respectively. These are considered high priority for surveillance efforts in order to confirm presence of these pathogens in counties classified as suitable.
Burtis 2023 [133]	Seasonal activity patterns of host-seeking Ixodes scapularis (Acari: Ixodidae) in Minnesota, 2015-2017	United States	To document the host-seeking activity of I. scapularis at 4 geographically distinct woodland sites throughout Minnesota to understand temporal risk for exposure to I. scapularis nymphs and adults and to assess the interannual and geographic variability in host-seeking phenology and determine the relative timing of the activity peaks for larvae and nymphs.	Tick Sampling	2015-2017	Adults were active throughout the entire 8-month collection season, with sporadic activity during the summer, larger peaks in activity observed in April, and lower peaks in October. Nymphs were most active from May through August, with continuing low-level activity in October, and peak activity in June.
Maxwell 2023 [105]	Improving Surveillance of Human Tick-Borne Disease Risks: Spatial Analysis Using Multimodal Databases	United States	To use multimodal databases, geographically overlaying patient survey data and assess the use of various indicators as proxies for human TBD risk.	Mixed methods	2000-2021	Thematic mapping results included one-for-one county-level matching of reported TBEs with at least 1 designated source of human disease risk (ie, positive canine serological tests, CDC-reported Lyme disease, or known tick presence).
Kopsco 2023 [147]	Current and Future Habitat Suitability Models for Four Ticks of Medical Concern in Illinois, USA	Illinois, USA	To assess the historical and future habitat suitability of four ticks of medical concern within the state, by fitting individual and mean-weighted ensemble species distribution models for Ixodes scapularis, Amblyomma americanum, Dermacentor variabilis, and a newly invading species, Amblyomma maculatum using a variety of landscape and mean climate variables.	Ensemble model projection	1970-2080	The suitable habitat for I. scapularis, A. americanum, and A. maculatum was predicted to significantly narrow in the 2050 climate scenario and then increase more broadly statewide in the 2070 scenario but at reduced likelihoods.

Maxwell 2023 [150]	Understanding Habitats and Environmental Conditions of White-Tailed Deer Population Density and Public Health Data to Aid in Assessing Human Tick-Borne Disease Risk	United States	To determine if deer population density aligns with official disease data at the county level from (1) positive canine serological reports for, anaplasmosis, and Lyme Disease (LD); (2) positive human cases of ehrlichiosis, anaplasmosis, LD, and Spotted Fever rickettsioses; and (3) tick infectivity.	Mixed effects modelling	2004-2016	Authors find similar spatial distributions between deer population density and human and canine TBDs in northeastern and southern Indiana, which are rural and mixed geographic areas.
MaysMaestas 2023 [151]	Ticks and Tick-Borne Pathogens from Wild Pigs in Northern and Central Florida	Northern and Central Florida, USA	To examine the use of tick collection from wild pigs in the state of Florida as a method of surveillance for ticks and tick-borne pathogens of human concern.	Tick surveillance	2019-2020	Landscape features associated with tick diversity and abundance, such as developed spaces, mixed forest, and shrub/scrub habitat were identified, and may be useful for identifying areas of increased risk of encounters with human-biting ticks.
Goddard 2023 [141]	Anomalous phenology of Ixodes scapularis (Ixodida: Ixodidae) in Mississippi	Mississippi, USA	To report an apparent sudden departure from the reported I. scapularis phenology, with 13 adult specimens collected from 9 widely separated counties in Mississippi.	Tick surveillance	2022-2022	The study found 13 adult I. scapularis from 9 widely separated areas in Mississippi which is remarkable, even enigmatic, especially in light of the fact that multiple tick surveys conducted in Mississippi over the last 40 year failed to ever produce even one specimen during that time frame.
